# Supplementary material for: Using Single-Case Experimental Design and Patient-Reported Outcome Measures to Evaluate the Treatment of Cancer-Related Cognitive Impairment in Clinical Practice
Source: Cancers (Basel). 2023 Sep 20;15(18):4643. doi: 10.3390/cancers15184643 (PMC10526413; doi:10.3390/cancers15184643)
Supplement: Supplementary file 1 [file cancers-15-04643-s001.zip › cancers-2585269-supplementary.pdf]

SUPPLEMENTAL MATERIALS

REDCap SCED Data Capture Project Model

The following steps are guidelines for development of a project in REDCap that can automatically administer PROMIS v2.0 Cognitive Function-Short Form 8a (CF8) for single case experimental design data capture:

(1) Using your institution's REDCap platform, create a new project with an appropriate title
(2) Under REDCap's Project Setup tab, enable the following modules under "Main Project Settings":
    a. "Use surveys in this project?"
    b. "Use longitudinal data collection with defined events?"
(3) Under REDCap's Project Setup tab, enable the following modules under "Enable optional modules and customizations"
    a. "Auto-numbering for records"
    b. "Scheduling module (longitudinal only)"
    c. "Display the Today/Now button for all date and time fields on forms/surveys"
(4) Under REDCap's Project Setup tab, define the following events using the "Define My Events" tab
    a. Event #1
        i. Days Offset: 0
        ii. Event Label: First Contact
    b. Event #2
        i. Days Offset: 1
        ii. Event Label: Demographics
    c. Event #3
        i. Days Offset: 2
        ii. Event Label: Time 1 Baseline (Assessment)
    d. Event #4
        i. Days Offset: 7
        ii. Event Label: Time 2 Baseline (NoMAAT)
    e. Event #5
        i. Days Offset: 14
        ii. Event Label: Time 3 Baseline (PreMAAT 1)
    f. Event #6
        i. Days Offset: 28
        ii. Event Label: Time 4 (PreMAAT 2)

g. Event #7
    i. Days Offset: 35
    ii. Event Label: Time 5 (PreMAAT 3)
h. Event #8
    i. Days Offset: 42
    ii. Event Label: Time 6 (PreMAAT 4)
i. Event #9
    i. Days Offset: 49
    ii. Event Label: Time 7 (PreMAAT 5)
j. Event #10
    i. Days Offset: 56
    ii. Event Label: Time 8 (PreMAAT 6)
k. Event #11
    i. Days Offset: 63
    ii. Event Label: Time 9 (PreMAAT 7)
l. Event #12
    i. Days Offset: 70
    ii. Event Label: Time 10 (PreMAAT 8)
m. Event #13
    i. Days Offset: 77
    ii. Event Label: Time 11 (Week 1 PostMAAT)
n. Event #14
    i. Days Offset: 120
    ii. Event Label: Time 11 (Month 1 PostMAAT)

(5) Under REDCap's Project Home and Design Tab, use Design to create the following forms
a. Email Entry
    i. This form will contain two fields; an auto-generated Record ID field, and a field called Email Address. This field must have Text Box as its type, and Email selected from the "Validation?" dropdown menu.
b. Demographics
    i. This form will contain any demographic information that will be collected, as well as a section for informed consent.
c. Electronic Questionnaire
    i. This form will contain the MAAT outcomes survey

(6) Under REDCap's Project Setup tab, enable the following module under "Enable optional modules and customizations" (may be listed under "Additional Customizations"
a. "Designate an email field for communications (including survey invitations and alerts)." When prompted to select the designated field, select the Email Address field from the Email Entry form.

(7) Under REDCap's Project Setup tab, select the "Designate Instruments for My Events" tab and match the following forms with the following events:
   a. Email Entry: First Contact
   b. Demographics: Demographics
   c. Electronic Questionnaire: Time 1 through Time 12

(8) Under REDCap's Project Home and Design Tab, navigate to the Designer tab, which will list the three forms. Enable Demographics and Electronic Questionnaire forms as surveys.

(9) For Demographics and Electronic Questionnaire survey settings, designate preferred Survey Title and Survey Instructions.

(10) Set up Automated Invitations for Demographics form. Condition for sending invitation should be When Email Entry form is completed at First Contact. Invitation should be sent immediately. Enable reminders, and specify for them to be sent every 1 day, up to 3 times. Enter preferred email subject and survey instructions. **Email body must include [survey-link].**

(11) Set up Automated Invitations for Electronic Questionnaire form. Enable reminders every 1 day up to 3 times for each timepoint, and enter preferred email subject and survey instructions for each timepoint, **email body must include [survey-link]**. Specifications at each timepoint should be as follows
   a. Time 1 Baseline (Assessment)
      i. Condition: Completion of Demographics Questionnaire at Demographics timepoint
      ii. Sent immediately
      iii. Instructions should ask respondent to complete before next MAAT visit
   b. Time 2 Baseline (NoMAAT)
      i. Condition: Completion of Electronic Questionnaire at Time 1 Baseline (Assessment)
      ii. Sent after time lapse of 7 days
      iii. Instructions should ask respondent to complete 7 days after initial clinical visit
   c. Time 3 Baseline (PreMAAT 1)
      i. Condition: Completion of Electronic Questionnaire at Time 2 Baseline (NoMAAT)
      ii. Sent after time lapse of 7 days
      iii. Instructions should ask respondent to complete on the same day but prior to the next visit with clinician
   d. Time 4 (PreMAAT 2)
      i. Condition: Completion of Electronic Questionnaire at Time 3 Baseline (PreMAAT 1)
      ii. Sent after time lapse of 7 days

   iii. Instructions should ask respondent to complete on the same day but prior to the next visit with clinician

e. Time 5 (PreMAAT 3)
   i. Condition: Completion of Electronic Questionnaire at Time 4 (PreMAAT 2)
   ii. Sent after time lapse of 7 days
   iii. Instructions should ask respondent to complete on the same day but prior to the next visit with clinician

f. Time 6 (PreMAAT 4)
   i. Condition: Completion of Electronic Questionnaire at Time 5 (PreMAAT 3)
   ii. Sent after time lapse of 7 days
   iii. Instructions should ask respondent to complete on the same day but prior to the next visit with clinician

g. Time 7 (PreMAAT 5)
   i. Condition: Completion of Electronic Questionnaire at Time 6 (PreMAAT 4)
   ii. Sent after time lapse of 7 days
   iii. Instructions should ask respondent to complete on the same day but prior to the next visit with clinician

h. Time 8 (PreMAAT 6)
   i. Condition: Completion of Electronic Questionnaire at Time 7 (PreMAAT 5)
   ii. Sent after time lapse of 7 days
   iii. Instructions should ask respondent to complete on the same day but prior to the next visit with clinician

i. Time 9 (PreMAAT 7)
   i. Condition: Completion of Electronic Questionnaire at Time 8 (PreMAAT 6)
   ii. Sent after time lapse of 7 days
   iii. Instructions should ask respondent to complete on the same day but prior to the next visit with clinician

j. Time 10 (PreMAAT 8)
   i. Condition: Completion of Electronic Questionnaire at Time 9 (PreMAAT 7)
   ii. Sent after time lapse of 7 days
   iii. Instructions should ask respondent to complete on the same day but prior to the next visit with clinician

k. Time 11 (Week 1 PostMAAT)
   i. Condition: Completion of Electronic Questionnaire at Time 10 (PreMAAT 8)
   ii. Sent after time lapse of 7 days

iii. Instructions should ask respondent to complete on the same day but prior to the next visit with clinician

l. Time 12 (Month 1 PostMAAT)

   i. Condition: Completion of Electronic Questionnaire at Time 11 (Week 1 PostMAAT)
   ii. Sent after time lapse of 30 days
   iii. Instructions should ask respondent to complete on the same day but prior to the next visit with clinician

(12) Test project thoroughly and then move into production
